# Use of Immunostaining for the diagnosis of Lymphovascular invasion in superficial Barrett’s esophageal adenocarcinoma

**DOI:** 10.1186/s12876-020-01319-7

**Published:** 2020-06-05

**Authors:** Isao Hosono, Ryoji Miyahara, Kazuhiro Furukawa, Kohei Funasaka, Tsunaki Sawada, Keiko Maeda, Takeshi Yamamura, Takuya Ishikawa, Eizaburo Ohno, Masanao Nakamura, Hiroki Kawashima, Takio Yokoi, Tetsuya Tsukamoto, Yoshiki Hirooka, Mitsuhiro Fujishiro

**Affiliations:** 1 Department of Gastroenterology and Hepatology, Nagoya University Graduate School of Medicine, 65 Tsurumai-cho, Showa-ku, Nagoya, 466-8550 Japan; 2 Department of Endoscopy, Nagoya University Hospital, Nagoya, Japan; 3 Department of Pathology, Nagoya University Hospital, Nagoya, Japan; 4 Department of Diagnostic Pathology, Fujita Health University School of Medicine, Toyoake, Aichi Japan

**Keywords:** Barrett’s esophageal adenocarcinoma, D2–40, CD31

## Abstract

**Background:**

The prevalence of Barrett’s esophageal adenocarcinoma (BEA) is increasing in Japan. Accurate assessment of lymphovascular invasion (LVI) after endoscopic resection or surgery is essential in evaluating treatment response. This study aimed to assess the usefulness of immunostaining in determining the extent of LVI in superficial BEA.

**Methods:**

We retrospectively included 41 patients who underwent endoscopic resection or surgery between January 2007 and July 2018. In all cases, 3-μm serial sections from paraffin-embedded resected specimens were used for hematoxylin and eosin (H-E) staining and immunostaining for D2–40 and CD31. Two specialized gastrointestinal pathologists (T.Y. and T.T.), blinded to clinical information, independently evaluated the extent of LVI from these specimens. The LVI-positivity rate was evaluated with respect to the depth of invasion, changes in the positivity rate on immunostaining, pathological characteristics of patients with LVI, lymph node metastasis or relapse, and course after treatment.

**Results:**

H-E staining alone identified LVI in 7 patients (positivity rate: 17.1%). Depths of invasion were categorized based on extension to the submucosa (SM) or deeper. On immunostaining for D2–40 and CD31, additional positivity was detected in 2 patients with SM1 and 1 SM3, respectively; LVI was detected in 10 patients (positivity rate: 24.4%). LVI-positivity rates with invasion of the superficial muscularis mucosa (SMM)/lamina propria mucosa (LPM)/deep muscularis mucosa (DMM), SM 1, 2, and 3 were 0, 75, 28.6, and 55.6%, respectively.

**Conclusions:**

Combined H-E staining and immunostaining is useful in diagnosing LVI in superficial BEA, particularly in endoscopically resected specimens.

## Background

In Barrett’s esophagus (BE), columnar epithelium replaces normal squamous epithelium in the distal esophagus owing to repeated esophageal inflammation, injury, and repair caused by regurgitation of gastric acid or bile [1, 2]. The longitudinal extension of Barrett’s mucosa covering the entire circumference of the esophagus for at least 3 cm and less than 3 cm is termed long-segment Barrett’s esophagus and short-segment Barrett’s esophagus, respectively [3]. Adenocarcinoma originating from BE is termed Barrett’s esophageal adenocarcinoma (BEA). In Europe and the US, BEA accounts for approximately 60% of all esophageal cancer cases [4], and recent reports suggest a rapid rise in incidence, exceeding that of esophageal squamous cell carcinoma [5]. Meanwhile, BEA is less frequent in Japan, comprising only 4.7% of all esophageal cancer cases [6]. However, the incidence of gastroesophageal reflux disease (GERD) has recently increased in Japan owing to the introduction of a Western-style diet and a decrease in the incidence of *Helicobacter pylori* infection [7]. This change may increase the incidence of BE, and consequently, BEA. Indeed, several studies have reported a slight increase in the incidence of BEA in Japan [8, 9]. The 5-year survival rate for advanced BEA without distant metastases is only < 20% [10]; thus, early diagnosis and treatment are essential.

Superficial BEA, in which the depth of cancer invasion is limited on submucosa, is primarily treated with surgery and endoscopic treatment as it has low risk for lymph node metastases. In Europe and the US, the primary treatment modality for BEA is endoscopic mucosal resection (EMR) combined with radiofrequency ablation (RFA) [11], while in Japan, the treatment involves endoscopic submucosal dissection (ESD) as en bloc resection. Additional treatment may be considered in cases extending to the deep muscularis mucosa (DMM) or deeper or with lymphovascular invasion (LVI). ESD, which facilitates en bloc resection, is more beneficial than EMR as it allows for fractional excision. ESD has been gradually introduced in Europe and the US [12].

However, given the rarity of BEA in Japan, no guidelines have been established for endoscopic resection of superficial BEA. Currently, endoscopic treatment is performed according to the guidelines for esophageal squamous cell carcinoma. With the increase in the number of indications for ESD, a multicenter cooperative study reported the possibility of expanding indications for ESD to superficial BEA. In the absence of both LVI and components of poorly differentiated carcinoma, lymph node metastases were not observed in BEA measuring ≤30 mm in the maximum diameter and in those with ≤500-μm infiltration to the SM. However, D2–40 or CD31/CD34 immunostaining was not performed to examine the presence of LVI. Furthermore, no central pathological diagnosis was obtained [13]. To date, no study has investigated the extent of LVI using immunostaining in superficial BEA treated by endoscopic resection or surgery. Therefore, we aimed to evaluate the use of immunostaining in identifying LVI in patients with superficial BEA.

## Methods

### Patients

This retrospective study evaluated 41 patients with superficial BEA who underwent endoscopic resection or surgery between January 2007 and July 2018 at the Nagoya University Hospital. Those treated at other hospitals and who received preoperative chemotherapy were excluded. Data on clinical information, endoscopic findings, treatments, histopathological findings, and course after treatment were collected from the electronic charts.

### Diagnoses

Pathological diagnoses were made according to the Japanese Classification of Esophageal Cancer 11th edition, published by the Japan Esophageal Society [3]. New muscularis mucosa can sometimes be found just below the columnar epithelium. In the Japanese classification of esophageal cancer, the primary muscularis mucosa is referred to as the deep muscularis mucosa (DMM), and the new muscularis mucosa is referred to as the superficial muscularis mucosa (SMM). Takubo et al. reported that the duplicated muscularis mucosa was found 71.6% of Barrett’s esophageal adenocarcinoma specimens resected endoscopically in German patients [14]. Japan Esophageal Society classified the depth of tumor invasion into 6 groups as follows:

SMM; Carcinoma in situ or tumor has invaded the superficial muscularis mucosa.

LPM; Tumor has invaded the lamina propria mucosa.

DMM; Tumor has invaded the deep muscularis mucosa.

**Fig. 1:**
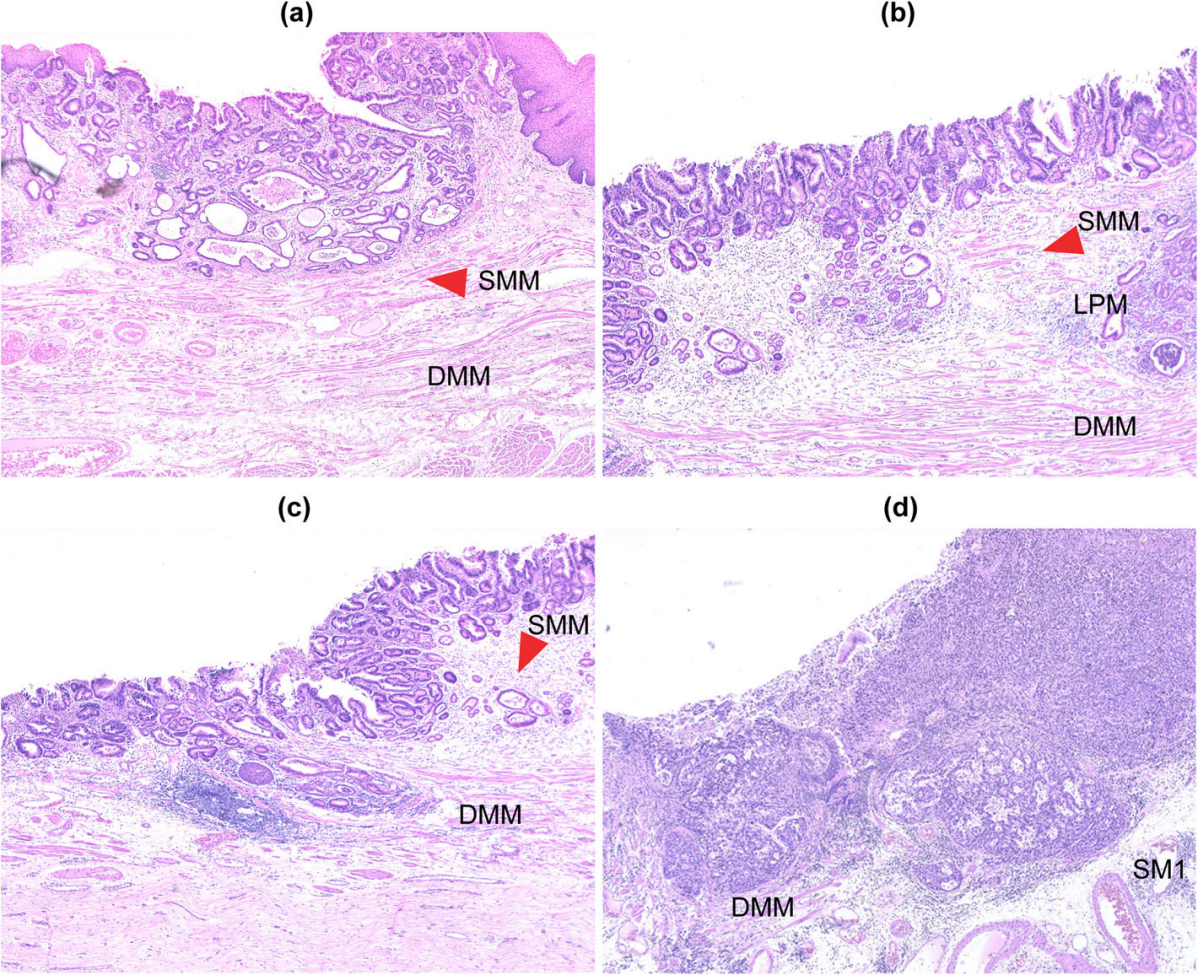
**a** Tumor invasion to SMM. **b** Tumor invasion to LPM. **c** Tumor invasion to DMM. **d** Tumor invasion to SM (SM1). The red arrowhead indicates SMM. Abbreviations: superficial muscularis mucosa (SMM), lamina propria mucosa (LPM), deep mucularis mucosa (DMM), submucosa (SM)

SM1; SM2; and SM3 involving ≤1/3 of the superficial, middle, and deep layers of the resected specimen, respectively (Fig. 1). Among the endoscopically resected specimens, those with an SM infiltration of ≤ and > 200 μm were regarded as SM1 and 2, respectively. Furthermore, SM infiltration cases were sub-divided into two groups based on a depth of SM infiltration of < and ≥ 500 μm, each of which were evaluated.

### Immunostaining and LVI assessment

To evaluate the presence of LVI, 3-μm serial sections were prepared from paraffin-embedded blocks of resected specimens. We used Podoplanin (D2–40) [15, 16] and CD31 [17] for immunostaining, which specifically stained the lymphatic and vascular endothelial cells, respectively. Of the serial sections, section 1 was stained with D2–40, section 2 with H-E, and section 3 with CD31. Immunostaining was performed by using an automated immunostainer and iView™ DAB (3,3′-diaminobenzidine) Detection Kit (Ventana Medical Systems, Inc., Tucson, AZ, USA) with labeled streptavidin biotinylated antibody methods. Antigen retrieval was performed by heat-induced epitope retrieval methods using a citrate buffer (pH 8.5) and a steamer at 100 °C for 60 min. The sections were immunostained with an antihuman D2–40 monoclonal antibody (clone D2–40, Dako), a mouse monoclonal antibody (JC70, Roche Tissue Diagnostics) for CD31. Counterstaining was performed using hematoxylin.

**Fig. 2:**
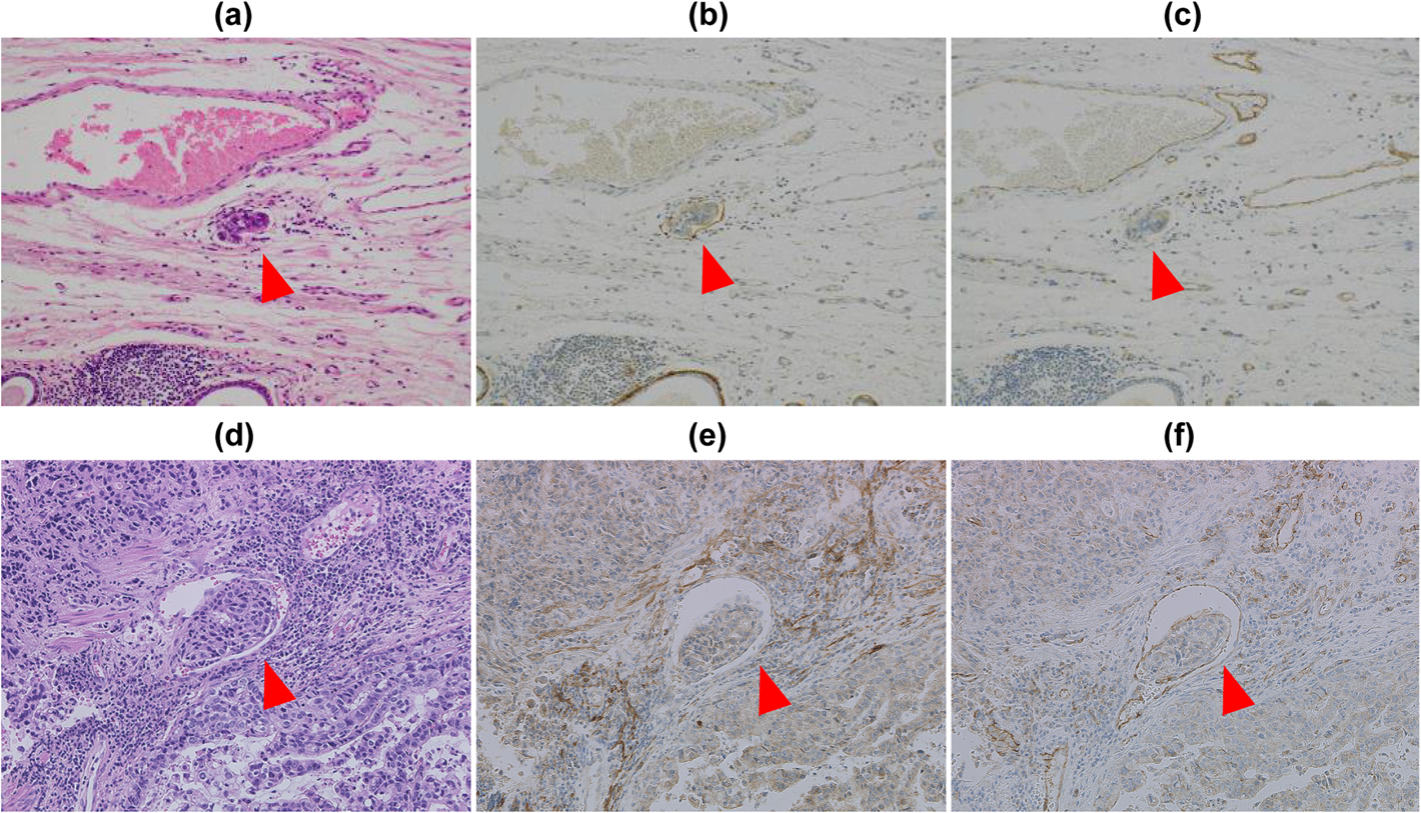
**a** Microphotograph of lymphovascular invasion (LVI) as assessed using hematoxylin and eosin (H-E) staining. **b** Microphotograph of lymphatic vessel invasion as assessed using D2–40 staining (positive). **c** Microphotograph of blood vessel invasion as assessed using CD31 staining (negative). **d** Microphotograph of lymphovascular invasion (LVI) as assessed by hematoxylin and eosin (H-E) staining. **e** Microphotograph of lymphatic vessel invasion as assessed by D2–40 staining (negative). **f** Microphotograph of blood vessel invasion as assessed by CD31 staining (positive)

LVI was microscopically assessed using the H-E- and immunostained specimens by two specialized gastrointestinal pathologists (T.Y. and T.T.) independently who were blinded to the clinical information. The evaluation of H-E and immunostained specimens were performed independently at different times (rather than simultaneously). LVI was defined as endothelial cells recognizable on D2–40- and CD31-positive cells and the presence of tumor cells in a space surrounded by these cells (Fig. 2).

The LVI-positivity rate was evaluated for the depth of invasion, changes in positivity rate on immunostaining, pathological characteristics of patients with LVI, lymph node metastasis or relapse, and treatment outcomes (overall, disease-specific, and relapse-free survival rates).

### Statistical analyses

Continuous and categorical variables were presented as median (region) and number (percentage), respectively. Clinical parameters were compared using the Mann-Whitney U test and Fisher’s exact test for continuous and categorical variables, respectively. The log-rank test was used to investigate the survival rate. A *p*-value of 0.05 was regarded as significant. All statistical analyses were performed using the IBM SPSS Statistics software version 25 (IBM SPSS, Chicago, IL, U.S.A.) package.

## Results

### Patient characteristics

The median age of the 41 patients was 67 years; the patient characteristics are detailed in Table 1. Macroscopically, protruding tumors were detected in 31 patients, and the median maximum tumor diameter was 20 mm. ESD and surgery were performed as initial treatments in 13 and 28 patients, respectively. The histological types in 21, 17, and 3 patients were well differentiated (tub1), moderately differentiated (tub2), and poorly differentiated (por), respectively. Clinicopathological characteristics did not differ significantly between patients with short-segment versus long-segment Barrett’s esophagus.

**Table 1:** Characteristics of patients treated by endoscopic submucosal dissection or surgery

Characteristics	All patients (*n* = 41)	SSBE^a^ (*n* = 30)	LSBE^b^ (*n* = 11)	SSBE VS. LSBE *P*-value
Age, median (range)	67 (39-81)	66 (39-88)	68 (44-79)	0.757
Sex (%)				
Male	32 (78.0)	23 (76.7)	9 (81.8)	1.000
Female	9 (22.0)	7 (23.3)	2 (18.2)	
Body mass index (kg/m^2^), median (range)	23.0 (16.7-32.6)	23.1 (16.7-32.6)	23.0 (16.7-32.6)	0.596
Tumor size (mm), median (range)	20 (6-60)	17.5 (6-35)	20 (10-60)	0.223
Macroscopic type (%)				
Protruding type	31 (75.6)	25 (83.4)	6 (54.5)	0.164
Flat type	2 (4.9)	1 (3.3)	1 (9.1)	
Depressed type	8 (19.5)	4 (13.3)	4 (36.4)	
Initial treatment (%)				
Endoscopic submucosal dissection (ESD)	13 (31.7)	12 (40.0)	1 (9.1)	0.127
Operation	28 (68.3)	18 (60.0)	10 (90.9)	
Histological type (%)				
Well differentiated (tub1)	21 (51.2)	18 (60.0)	4 (36.4)	0.181
Moderately differentiated (tub2)	17 (41.5)	11 (36.7)	5 (45.4)	
Poorly differentiated (por)	3 (7.3)	1 (3.3)	2 (18.2)	

^a^*SSBE* short-segment Barrett’s esophagus

^b^*LSBE* long-segment Barrett’s esophagus

### Histopathological findings

Table 2 shows the histological type and number of patients with LVI on H-E staining and immunostaining for D2–40/CD31 according to the depth of invasion. Overall, 21 and 20 patients had pT1a and pT1b lesions, respectively, and 12 of the 21 patients with pT1a had DMM lesions. The depth of SM infiltration in endoscopic resection exceeded 200 μm in 3 patients, and the depths were 400, 800, and 1300 μm, respectively. Among them, 2 patients underwent additional surgery, which revealed no residual cancer or lymph node metastases. The remaining one patient opted not to have surgery and instead was evaluated on follow-up; subsequently, she had no recurrence in 3-years following ESD. The incidences of histological subtypes with tub2 and por increased as invasion increased.

**Table 2:** Histological Characteristics with Respect to Depth of Invasion, and Comparison of LVI-positivity Rates between H-E- and D2-40-/CD31-stained Specimens

Depth of invasion and number			Histological type			H-E staining			Immunostaining			*P*-value
			tub1	tub2	por	Ly+	V+	LVI+ (%)	D2-40 Ly+	CD31 V+	LVI+ (%)	
T1a	SMM	7	6	1	0	0	0	0 (0)	0	0	0 (0)	
	LPM	2	1	1	0	0	0	0 (0)	0	0	0 (0)	
	DMM	12	10	2	0	0	0	0 (0)	0	0	0 (0)	
T1b	SM1	4	1	3	0	1	0	1 (25)	3	0	3 (75)	
	SM2	7	1	5	1	2	1	2 (28.6)	2	2	2 (28.6)	
	SM3	9	2	5	2	4	2	4 (44.4)	4	2	5 (55.6)	
Total		41						7 (17.1)			10 (24.4)	0.587

*H-E* Hematoxylin and eosin, *SMM* superficial muscularis mucosa, *LPM* lamina propria, *DMM* deep muscularis mucosa, *SM* submucosa

**Fig. 3:**
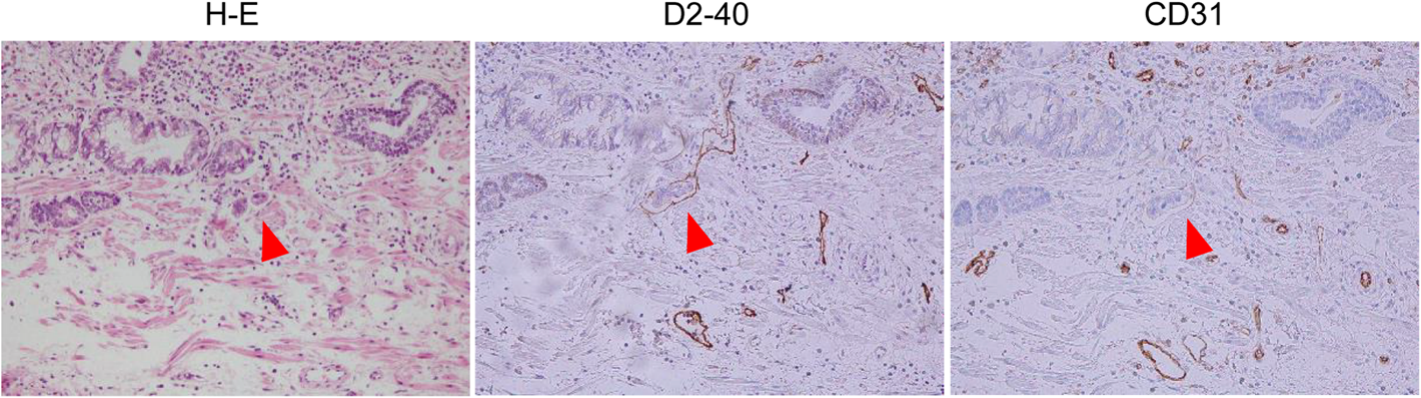
A case where immunostaining was useful for evaluating the presence of lymphovascular invasion (patient with SM1, in whom the lymphatic endothelial cells were very thin, making recognition difficult. Lymphovascular invasion was detected on immunostaining for D2–40). Abbreviations: submucosa (SM)

**Fig. 4:**
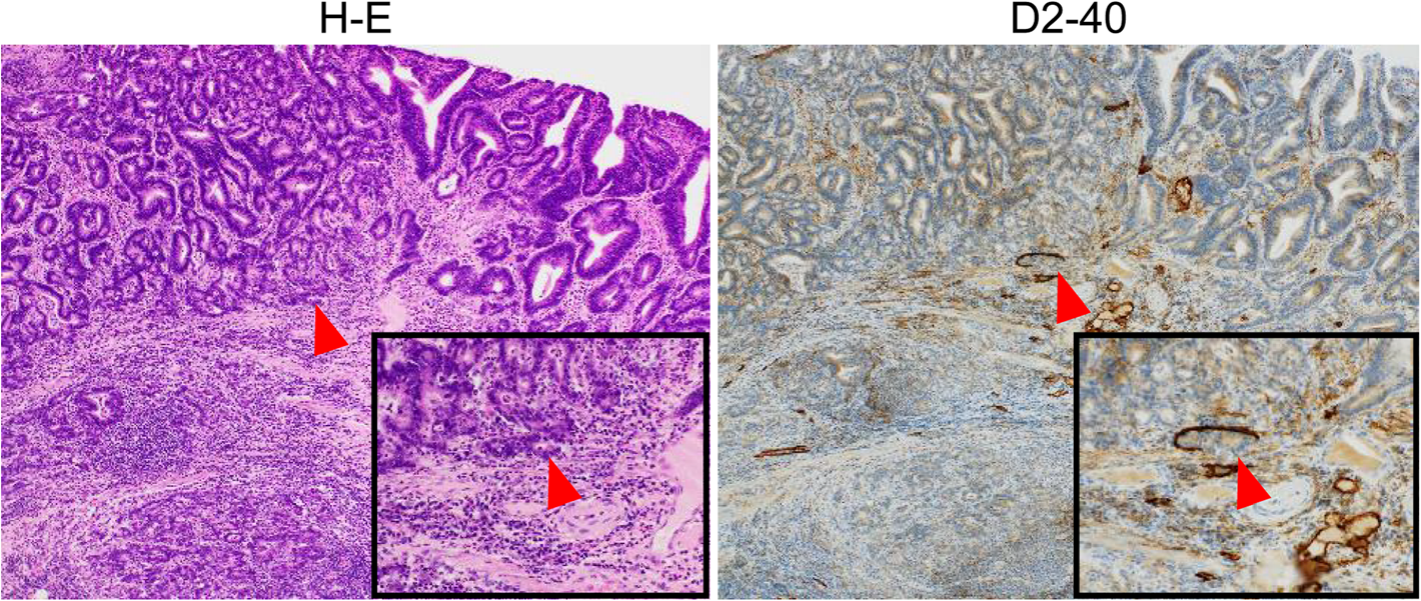
A case where immunostaining was useful for evaluating the presence of lymphovascular invasion (patient with SM3, in whom the tumor volume was large, making the assessment of lymphovascular invasion at the site of tumor infiltration impossible. Lymphovascular invasion was detected on immunostaining with D2–40). Abbreviations: submucosa (SM)

In 7 patients, LVI positivity was noted using H-E-stained specimens alone (positivity rate: 17.1%), and the depth of invasion was evaluated to be SM1 or deeper. LVI was found in 10 patients (positivity rate: 24.4%) who were additionally diagnosed with LVI positivity on immunostaining for D2–40 and CD31. The concordance rate of LVI diagnosis between the two pathologists was 97.6% (40/41) for H-E-stained specimens and 92.7% (38/41) for immunostained specimens. The kappa coefficient for the two pathologists was 0.92 for H-E-stained specimens and 0.82 for immunostained specimens, which indicated almost perfect agreement. The LVI-positivity rates in SMM, LPM, DMM, SM1, SM2, and SM3 lesions were 0, 0, 0, 75, 28.6, and 55.6%, respectively. Overall, between H-E staining alone and immunostaining, LVI was consistently absent in 75.6% (31/41) cases. LVI was additionally detected on immunostaining in cases with SM1 (Fig. 3), in which the lymphatic endothelial cells were very thin near the tumor margin (site where LVI diagnosis is relatively easy), making recognition difficult, and in cases with SM3 (Fig. 4), in which the tumor volume was large, making the identification of LVI at the site of tumor infiltration impossible.

The patients in the SM group were sub-divided into two groups based on the depth of infiltration as follows: < 500 μm and ≥ 500 μm. The former subgroup had 5 patients with SM1 lesions (4 and 1 underwent surgery and endoscopic resection with a depth of infiltration of 400 μm, respectively). The latter subgroup included 15 patients. LVI was present in 3 (60%) of the patients with < 500 μm submucosal infiltration and in 7 (46.7%) of patients with ≥ 500 μm submucosal infiltration. No specific pattern of distribution of LVI sites was observed.

### Lymph node metastasis and relapse

Table 3 shows the pathological findings in 30 surgically treated patients with superficial BEA, who underwent surgical treatment, including 2 patients who underwent additional treatment after ESD. In total, 3/41 (7.3%) patients showed lymph node metastases. All three patients had protruding cancers derived from SSBE, invading at least up to SM2 (depth of infiltration: > 1000 μm). The tumor maximal diameters were ≥ 25 mm, and they contained poorly differentiated components. LVI was identified in 2 of 3 patients.

**Table 3:** Pathological Findings in 30 Patients with Superficial Cancer who Underwent Surgery

Depth of invasion	Number of patients	Histological type			Lymphovascular invasion	Lymph node metastasis	Recurrence
		tub1	tub2	por	LVI+	+	+
SMM	3	3	0	0	0	0	0
LPM	2	1	1	0	0	0	0
DMM	6	5	1	0	0	0	0
SM1	4	1	3	0	3	0	0
SM2	6^*^	1	4	1	2	2	2
SM3	9	3	4	2	5	1	1
Total	30	14	13	3	10	3	3

^*^Including 2 patients who underwent additional surgery after ESD

*SMM* superficial muscularis mucosa, *LPM* lamina propria, *DMM* deep muscularis mucosa, *SM* submucosa

### Overall survival rate and relapse-free survival

**Fig. 5:**
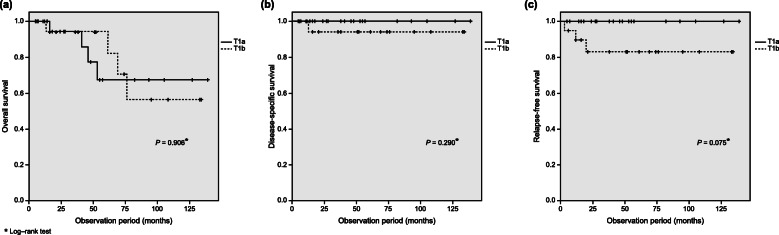
Survival curves in patients with T1a/T1b tumors. **a** Overall survival. **b** Disease-specific survival. **c** Relapse-free survival

The recurrence rate was slightly higher among patients with T1b disease. However, there were no significant differences in overall, disease-specific, and relapse-free survival between patients with T1a and T1b disease (Fig. 5). Relapse occurred in 3 patients with T1b disease, with a median follow-up of 46 months. In all 3 patients, LVI was present, the depth of invasion was evaluated to be at least SM2, poorly differentiated components were observed, and the tumor diameter was ≥20 mm. Among them, 1 patient died of primary disease. The 3-year disease-specific survival rate in those with T1a and T1b disease was 100 and 95.0%, respectively.

## Discussion

The results of this study show that combined H-E staining and immunostaining is useful in diagnosing LVI in superficial BEA, particularly in endoscopically resected specimens. LVI is directly related to lymph node/remote metastases in cancer patients [18–22]. Therefore, LVI may be useful in predicting the metastasis risk. In Japan, few studies have reported on the incidence of LVI positivity in BEA patients. Osumi et al. identified LVI in 18/55 lesions (32.7%) with DMM [23]. Furthermore, Nishi et al. observed in lymphatic invasion in 10.3% of cases with DMM invasion. Further, also reported that the LVI-positivity rate increased with the depth of invasion [8].

In this study, LVI was present in patients with depths of invasion of at least SM1. The differences from previous reports were probably due to the number of patients and the use of immunostaining to identify LVI in all patients. Additional immunostaining increased the LVI-positivity rate by 7% than H-E staining alone. In patients with SM1 lesions, this rate increased from 25 to 75%. Although the number of SM1 patients was small (*n* = 4), the high positivity rate is noteworthy. LVI is usually assessed using H-E-stained specimens. In patients in whom assessment is exceptionally difficult, the results may depend on the pathologist’s subjective assessment [24, 25]. Particularly, it is difficult to evaluate fine lymphatic/venous invasion; difficult-to-identify lymphovascular endothelial cells; desmoplastic reaction of interstitial cells [26–28]; and artifacts related to tissue specimen preparation [25, 29, 30]. Here, LVI diagnosis was also difficult in some patients. Particularly, the difficulty in recognizing lymphatic/blood vessels may increase with a reduction in the grade of tumor differentiation. These factors limit LVI assessment using H-E-stained specimens alone. Additional immunostaining may have increased the LVI-positivity rate among SM infiltrating lesions in this cohort.

Japanese guidelines recommend endoscopic treatment for early esophageal cancer and early gastric cancer. Conversely, no treatment guidelines for BEA have been developed owing to lack of data. In this study, LVI was absent in patients with infiltration up to the DMM. In SM1 lesions, no lymph node metastases were observed when the criteria proposed by Ishihara et al. were fulfilled [13]. This suggests that ESD may be increasingly employed in these cases. Notably, LVI, invasion to SM2 or deeper, presence of poorly differentiated components, and a maximum tumor diameter of ≥20 mm were common among patients with relapse. The patients with SM or superficial lesions had relatively favorable prognosis, and only few patients had relapse. Therefore, the risks of lymph node metastasis and relapse may be low in SM (infiltration: < 500 μm) lesions with a maximum diameter of ≤20 mm, absence of LVI, and absence of poorly differentiated carcinoma components. This suggests that after ESD, follow-up is a feasible option in patients ineligible for surgery. However, LVI is detected on immunostaining in some patients with SM1 invasion. Therefore, pathological findings should be carefully evaluated with additional immunostaining.

The risk of lymph node metastasis must be adequately evaluated. Many studies reported that LVI, detected on immunostaining for D2–40, was an independent prognostic factor for lymph node metastasis [20, 21, 31, 32]. LVI diagnosis may predict subsequent lymph node metastasis. In this study, only few patients had lymph node metastasis or relapse, making detailed statistical analysis unreliable.

As a result of immunostaining, LVI was newly diagnosed in some patients and ruled out in some cases despite positivity on H-E staining. Although we were unable to conclude statistically whether additional immunostaining significantly increased the LVI-positivity rate in comparison with H-E staining alone, immunostaining may be useful in individual patients. The LVI-positivity rate was high among those with SM1 invasion; this should be considered while selecting patients for ESD. Furthermore, it is important to be able to identify the presence of LVI for predicting future relapse in patients with SM2 and SM3. In addition, positive findings on additional immunostaining in endoscopically resected specimens may facilitate decision-making for further treatment and prevent unnecessary surgery. However, immunostaining is cost and effort intensive and should be considered carefully in limited-resource settings.

The limitations of this study are the single-center retrospective design and small sample size. However, immunostaining for D2–40 and CD31 was performed in all patients with superficial BEA who underwent ESD or surgery, and the presence of LVI was examined. Furthermore, the proportion of surgically treated patients was relatively high; the number of evaluable cases with lymph node metastases was also large.

## Conclusion

Immunostaining for D2–40 and CD31 is useful for identifying the presence of LVI in patients with superficial BEA. This is essential for evaluating the need for additional treatment, particularly in endoscopically resected specimens. Prospective multicenter studies on ESD and surgery as treatment options for superficial cancer, are needed.

## Data Availability

The datasets used and/or analyzed during the current study are available from the corresponding author on reasonable request.

## Abbreviations

SMM: superficial muscularis mucosa
LPM: lamina propria mucosa
DMM: deep muscularis mucosa
SM: submucosa
BEA: Barrett’s esophageal adenocarcinoma
BE: Barrett’s esophagus
ESD: endoscopic submucosal dissection
